# Fulminant Multidrug-Resistant *Streptococcus pneumoniae* Meningoencephalitis After Elective ENT Surgery: A Case Report

**DOI:** 10.3390/microorganisms13102315

**Published:** 2025-10-07

**Authors:** Corina-Ioana Anton, Madalina Maria Zamfir, Alexandru Ghiță, Mihaela Raluca Mititelu

**Affiliations:** 1 Faculty of General Medicine, Carol Davila University of Medicine and Pharmacy, 8 Eroii Sanitari Bvd, 050474 Bucharest, Romania; corina-ioana.anton@drd.umfcd.ro (C.-I.A.); zamfir.madalinamaria@yahoo.com (M.M.Z.); 2 Department of Medico-Surgical and Prophylactic Disciplines, Titu Maiorescu University, 040441 Bucharest, Romania; 3 Department of Infectious Diseases, “Dr. Carol Davila” Central Military Emergency University Hospital, 134 Calea Plevnei, 010242 Bucharest, Romania; 4 Medical Informatics Office, Telemedicine, Communications and Information Technology Agency, 134 Calea Plevnei, 010242 Bucharest, Romania; 5 Department of Nuclear Medicine, University of Medicine and Pharmacy Carol Davila, 020021 Bucharest, Romania; 6 Department of Nuclear Medicine, University Emergency Central Military Hospital, 010825 Bucharest, Romania

**Keywords:** pneumococcal meningitis, antimicrobial resistance, septoturbinoplasty, vaccination, ENT surgery

## Abstract

Pneumococcal meningoencephalitis is a severe infection associated with high morbidity and mortality. Although typically community-acquired, postoperative cases following elective ENT surgery are exceedingly rare. Antimicrobial resistance (AMR) among *Streptococcus pneumoniae* further complicates management, and missed opportunities for vaccination represent preventable risks. We report a case of a 41-year-old man with multiple comorbidities who developed fulminant *S. pneumoniae meningitis* 48 h after septoturbinoplasty. The clinical course was atypical, with altered consciousness but no classical meningeal signs, necessitating urgent intubation and intensive care admission. Cerebrospinal fluid cultures identified an MDR pneumococcal strain resistant to penicillin and macrolides but susceptible to vancomycin and meropenem. Empirical therapy with vancomycin and meropenem, combined with adjunctive corticosteroids and multidisciplinary ICU care, led to complete neurological recovery. This case highlights a rare but life-threatening postoperative complication and underscores two critical lessons. First, the growing challenge of multidrug-resistant pneumococcus requires timely recognition, aggressive empiric therapy, and access to effective agents. Second, the absence of pneumococcal vaccination in this high-risk surgical patient illustrates a preventable gap in care. Integrating vaccination screening into preoperative evaluations may reduce the risk of catastrophic postoperative CNS infections.

## 1. Introduction

*Streptococcus pneumoniae*, a Gram-Positive bacterium, is a leading cause of bacterial meningitis and meningoencephalitis worldwide. Its ability to invade the central nervous system (CNS) and cause severe inflammation of the meninges and brain tissue poses a significant health risk. Pneumococcal meningoencephalitis is a relatively common community-acquired infection, but its occurrence following septoturbinoplasty is exceptionally rare and potentially life-threatening [1].

Surgical procedures that involve manipulation of the nasal structures can inadvertently create pathways for bacterial invasion by disrupting the natural mucosal barriers that typically protect against pathogen entry [2]. Pneumococcal meningoencephalitis after septoturbinoplasty can occur through two primary mechanisms. First, the direct disruption of mucosal barriers during surgery can provide a route for the contiguous spread of bacteria from the upper respiratory tract to the CNS. Second, the transient immune suppression that often follows surgical procedures can compromise the body’s ability to combat invading pathogens, allowing hematogenous dissemination [2,3].

Postoperative pneumococcal meningoencephalitis presents unique diagnostic and therapeutic challenges due to its atypical clinical presentation and rarity in otorhinolaryngological (ENT) procedures such as septoturbinoplasty [2]. Unlike classic community-acquired cases, postoperative infections may not always manifest with typical meningeal signs, often resulting in delayed diagnosis and treatment. This underscores the challenges in diagnosing and managing such rare complications, emphasizing the need for a high index of suspicion and rapid intervention to improve patient outcomes [3].

The pathogenesis involves a complex interplay between surgical trauma, host immune response, and microbial virulence. Surgical manipulation of the highly vascularized nasal mucosa can induce micro-perforations and transient disruption of epithelial barriers, facilitating bacterial translocation into deeper tissues [4]. Concurrently, surgical stress induces immunomodulatory effects, including lymphopenia and impaired neutrophil chemotaxis, which weaken early innate immune defenses [4]. Furthermore, comorbidities such as diabetes mellitus, hypertension, and vascular disease exacerbate immune dysfunction and compromise blood–brain barrier integrity, increasing susceptibility to invasive pneumococcal disease [4,5].

The rising incidence of multidrug-resistant *S pneumoniae* strains complicates empirical antimicrobial therapy, underscoring the need for agents with robust cerebrospinal fluid penetration and broad-spectrum coverage. Adjunctive corticosteroid therapy remains essential to reduce inflammatory-mediated neurological damage and improve patient outcomes [5].

This case report highlights the importance of vigilance in postoperative care and the need for prompt recognition and treatment of CNS infections following nasal surgery.

## 2. Case Presentation

A 41-year-old man with a complex past medical history including bilateral pulmonary embolism, right temporo-parietal hemorrhagic stroke, recurrent urinary tract infections (UTIs) with *Pseudomonas aeruginosa*, type 2 diabetes mellitus, arterial hypertension, dyslipidemia, and benign prostatic hyperplasia (BPH), was admitted to the Emergency Department of “Dr. Carol Davila” Central Military Emergency University Hospital in Bucharest Romania on 24 March 2025.

His recent surgical history included elective septoturbinoplasty performed 48 h prior. Routine preoperative evaluation included complete blood count, serum chemistry, coagulation profile, and nasal endoscopy, all of which were within normal limits. No signs of active infection were observed. Standard nasal antisepsis with povidone–iodine was performed. Intravenous Cefazolin (1 g) was administered 60 min before the incision as prophylaxis, in accordance with the institutional ENT protocol. No pneumococcal vaccination was administered prior to surgery.

Septoturbinoplasty was performed under general anesthesia using an endoscopic technique. A left hemitransfixion incision was made, bilateral mucoperichondrial flaps were elevated, and the deviated septal cartilage and bony spurs were removed using a microdebrider and Blakesley forceps. The inferior turbinates were reduced using submucosal radiofrequency ablation. The total operative time was approximately 60 min. No dural exposure, skull base defects, or cerebrospinal fluid leaks were observed. Silicone splints and standard hemostatic nasal packing were placed and removed after 24 h. The immediate postoperative course was uneventful.

He presented with fever (38.6 °C), chills, left-sided otalgia, and progressive alteration in mental status that developed over the preceding 24 h. Upon arrival, the patient was agitated and exhibited progressive confusion, necessitating analgosedation, orotracheal intubation, and mechanical ventilation for airway protection and to prevent neurological deterioration.

Upon physical examination, the patient was sedated and received invasive ventilatory support. The pupils were symmetrical and reactive to light; however, the oculocephalic reflex was absent. No neck stiffness was observed initially. Skin examination revealed minor scratched lesions on the left thigh. Cardiopulmonary examination was unremarkable, with stable hemodynamic and respiratory parameters. The abdomen was soft and nontender. Neurological examination revealed unresponsiveness to painful stimuli and symmetrically dropped limbs, consistent with diffuse encephalopathy.

Given his immunometabolic vulnerability and complex vascular history, a rapid diagnostic workup and empirical management were initiated due to a high clinical suspicion of a central nervous system (CNS) infection, with subsequent findings confirming pneumococcal meningoencephalitis.

The pathogen was identified Via cerebrospinal fluid culture and confirmed using the VITEK 2 automated system (bioMérieux). Antimicrobial susceptibility testing revealed a multidrug-resistant profile: the strain was resistant to penicillin, macrolides, and trimethoprim-sulfamethoxazole, but susceptible to vancomycin, meropenem, fluoroquinolones, and third-generation cephalosporins. Based on this profile, the patient was initiated on empirical intravenous therapy with meropenem and vancomycin, both of which offer reliable cerebrospinal fluid penetration and broad-spectrum coverage suitable for resistant pneumococcal infections. Blood cultures were collected within one hour prior to the initiation of antibiotic therapy, and the results were negative.

According to EUCAST 2025 interpretive criteria, the isolate was resistant to penicillin (MIC ≥ 2 µg/mL), erythromycin (MIC ≥ 1 µg/mL), and trimethoprim-sulfamethoxazole (MIC ≥ 4/76 µg/mL), and susceptible to vancomycin (MIC ≤ 0.5 µg/mL), meropenem (MIC ≤ 0.25 µg/mL), levofloxacin (MIC ≤ 2 µg/mL), and ceftriaxone (MIC ≤ 0.5 µg/mL).

Preoperative laboratory tests performed two days before surgery were within normal limits. Laboratory investigations performed on admission (postoperative day 2), approximately 48 h after surgery show systemic inflammation, metabolic disturbances, and mild liver injury (Table 1). Arterial blood gas analysis confirmed acidosis with a low bicarbonate level, normal pCO_2_, and elevated lactate levels, consistent with metabolic acidosis and tissue hypoperfusion (Table 2).

As shown in Table 3, CSF analysis summarizes the key laboratory findings. The appearance was opalescent, consistent with bacterial meningitis, contrasting with the clear appearance expected in a normal sample. Opening pressure was elevated at 200 mmH_2_O (reference range: 70–180 mmH_2_O). Cell count analysis revealed marked pleocytosis with a predominance of polymorphonuclear leukocytes. Glucose concentration was markedly reduced to 0 mg/dL, while sodium and potassium levels remained within the normal CSF ranges. Notably, albumin and total protein levels were significantly elevated (83 mg/dL and 283 mg/dL, respectively). Microbiological culture confirmed the presence of *S. pneumoniae* with a multidrug-resistant (MDR) profile.

CSF analysis revealed marked pleocytosis, with a total leukocyte count of 1850 cells/mm^3^, comprising 92% polymorphonuclear leukocytes and 8% lymphocytes, findings consistent with acute bacterial meningitis.

Pronounced neutrophilic predominance indicates a fulminant inflammatory response.

Neuroimaging—including CT and subsequent MRI—demonstrated mild sinus opacification and mastoid involvement, without evidence of parenchymal lesions or thrombosis (Table 4).

**Figure 1:**
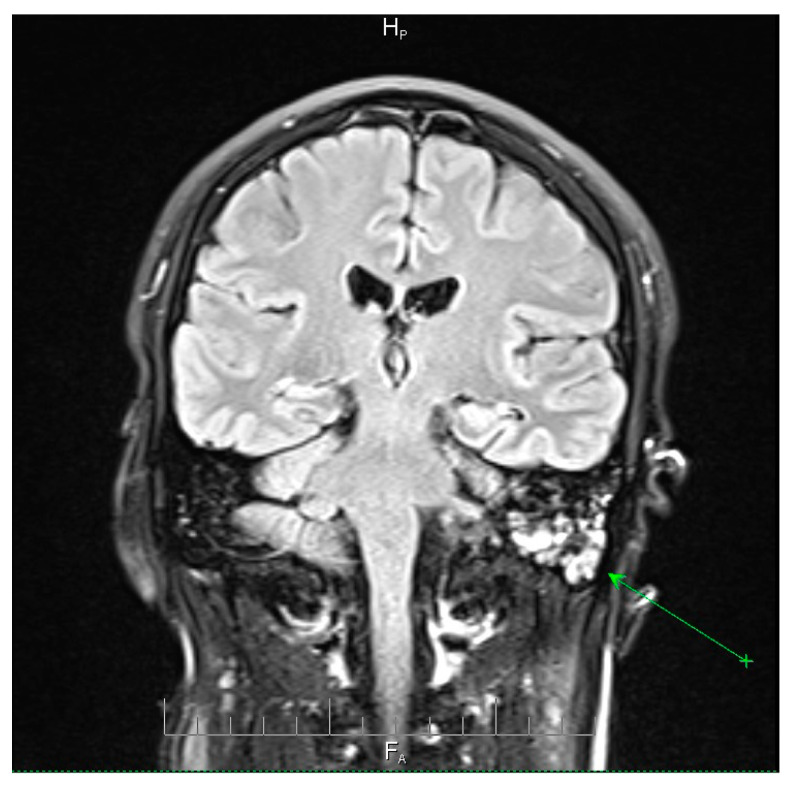
Coronal T2-weighted MRI scan demonstrating para-fluid collections within the left mastoid air cells extending toward Citelli’s angle (green arrow). There was no evidence of parenchymal lesions, intracerebral edema, or venous sinus thrombosis. The surrounding brain parenchyma and the ventricular system appeared normal. These findings are consistent with localized mastoid involvement and help exclude intracranial complications, providing an important radiological context for the patient’s clinical presentation. H_p_ = head-posterior (shows the orientation of image). F_A_ = foot-anterior (indicates the directional axis at the bottom of the image).

**Figure 2:**
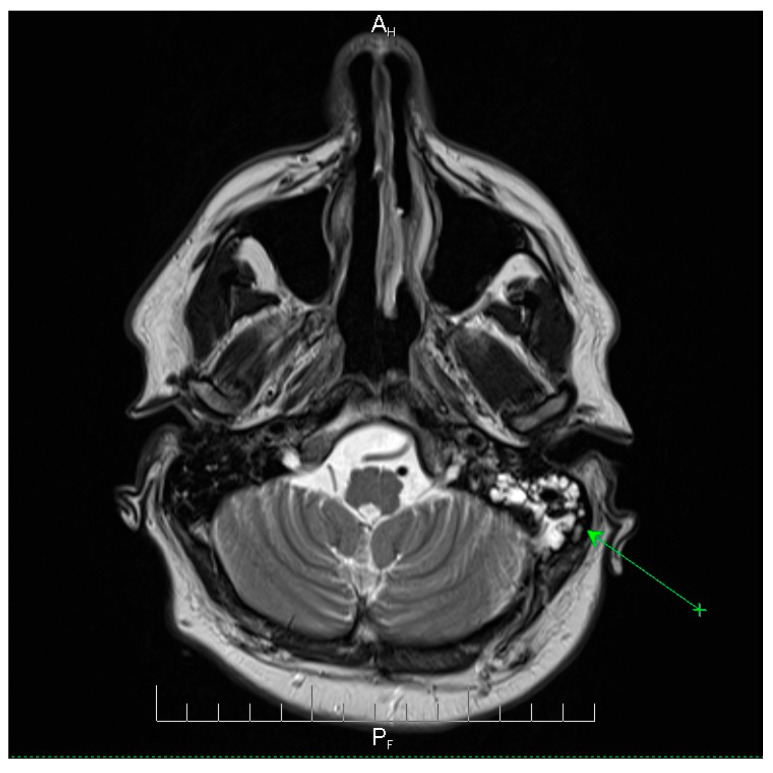
Axial T2-weighted MRI scan demonstrating opacification of the left mastoid air cells with a hyperintense signal (green arrow), indicative of mastoid involvement. This finding suggests a potential contiguous source of intracranial infection in this patient. No acute lesions, hemorrhage, or signal abnormalities were observed in the cerebellum or brainstem, and the surrounding brain parenchyma appeared normal. These imaging features support localized mastoid pathology without evidence of intracranial extension, providing important radiological context for the patient’s clinical presentation and aiding in the assessment of the infection risk. A_H_ = anterior-head. P_F_ = posterior- foot.

**Figure 3:**
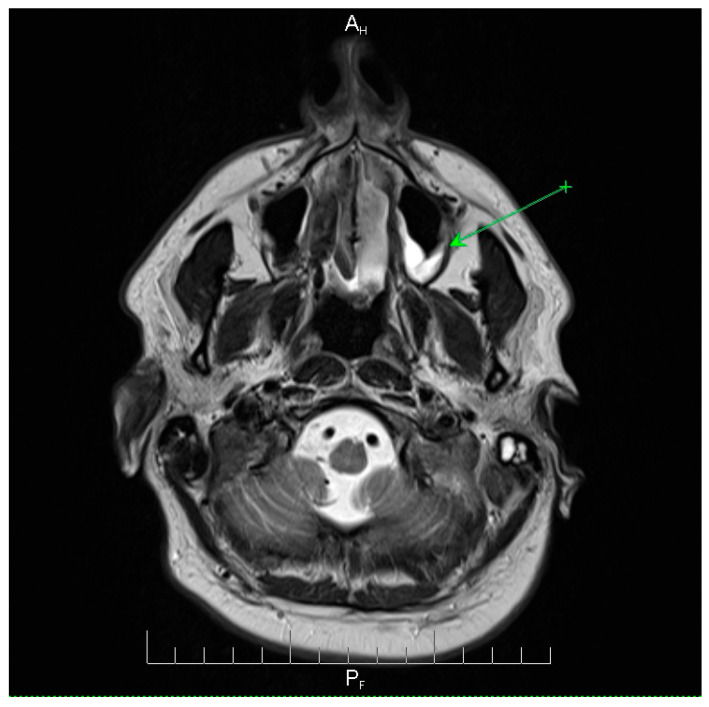
Axial T2-weighted MRI scan demonstrating the post-septoturbinoplasty reduction status on the right. Mild inflammatory changes were observed in the mucosa of the maxillary sinuses, predominantly on the left side, with less pronounced involvement of the ethmoidal cell system. No evidence of acute intracranial pathology or parenchymal lesions was observed. These findings indicate residual postoperative changes and mild sinonasal inflammation, providing context for the patient’s clinical presentation and helping to differentiate postoperative alterations from active infectious processes. A_H_ = anterior-head. P_F_ = posterior- foot. Axial T2-weighted MRI scan demonstrating the post-septoturbinoplasty reduction status on the right. The green arrow indicates a focal area of mucosal thickening and fluid signal within the left maxillary sinus, consistent with mild inflammatory changes, with less pronounced involvement of the ethmoidal cell system. No evidence of acute intracranial pathology or parenchymal lesions was observed. These findings indicate residual postoperative changes and mild sinonasal inflammation, providing context for the patient’s clinical presentation and helping to differentiate postoperative alterations from active infectious processes.

Neuroimaging revealed mild ethmoidal/sphenoidal sinus opacification and left mastoid air cell involvement without evidence of parenchymal lesions or venous sinus thrombosis (Figure 1, Figure 2 and Figure 3). Coronal and axial T2-weighted MRI sequences revealed para-fluid collections within the left mastoid air cells extending toward Citelli’s angle (Figure 1, Figure 2 and Figure 3), consistent with an otogenic focus and a possible contiguous route for intracranial spread.

The patient was started on empirical intravenous meropenem 2 g every 8 h and vancomycin 1 g every 8 h. Adjunctive corticosteroids (dexamethasone) were administered to reduce meningeal inflammation. Sedation with haloperidol and alprazolam was initiated for agitation control. Supportive therapies included anticoagulation, gastric mucosal protection and vitamin supplementation. Oral candidiasis was treated with fluconazole, while a suspected urinary tract infection was managed with trimethoprim-sulfamethoxazole (TMP-SMX). Mechanical ventilation and intensive care unit-level care were provided.

The patient underwent a 14-day course of antibiotic therapy specifically targeting the pneumococcal infection, resulting in a gradual improvement in clinical condition and resolution of symptoms.

Neurological and systemic improvements were observed for 10 days. Repeat lumbar puncture (9 April 2025) showed normalized CSF parameters and reduced cellularity (18 mononuclear cells). The patient was extubated, fully conscious, cooperative, and hemodynamically stable. The discharge medications included oral TMP-SMX and local intranasal therapy. The patient was vaccinated post-discharge with PCV15 (pneumococcal conjugate vaccine), to be followed by PPSV23 (polysaccharide vaccine) after one year, as per CDC guidelines for high-risk adults. He also received the quadrivalent MenACWY conjugate vaccine for *Neisseria meningitidis* and was advised annual influenza vaccination.

## 3. Discussions

Postoperative meningitis after sinonasal surgery is exceptionally rare, with reported rates of < 0.1% across large surgical series [6]. Among these, fulminant multidrug-resistant (*MDR*) *S. pneumoniae* infection within 48 h of an otherwise uncomplicated septoturbinoplasty has scarcely been reported [6]. Therefore, this case expands the limited surgical literature by documenting a fulminant postoperative course, early imaging evidence of mastoid involvement suggesting contiguous spread, and the preventive implications of pneumococcal vaccination in high-risk ENT patients.

Compared to previously documented cases of postoperative meningitis, which typically arise after surgeries involving direct skull base exposure or cerebrospinal fluid leakage, this case presents several unique aspects [3,5]. First, the initial surgery was a standard septoturbinoplasty conducted without any intraoperative dural breach. Second, the responsible organism was a multidrug-resistant *S. pneumoniae*, whereas most postoperative meningitis cases are linked to Gram-Negative rods or methicillin-susceptible *S. aureus*. Third, the lack of pneumococcal vaccination in high-risk adults underscores a preventable factor that has not been highlighted in earlier reports. Together, these characteristics enrich the surgical literature and offer new clinical insights into multidrug-resistant pneumococcal disease following low-risk ENT surgery.

Our patient developed clinically overt disease within 48 h of surgery, highlighting that even low-risk otorhinolaryngological procedures can precipitate life-threatening intracranial complications under conducive host and microbial conditions. The correlation between laboratory and imaging data suggests a synchronous systemic and CNS inflammatory response. Elevated CRP, profound leukocytosis, and metabolic acidosis reflect systemic inflammation, whereas CSF analysis reveals the classical profile of acute bacterial meningitis—opalescent appearance, neutrophilic pleocytosis, hypoglycorrhachia, and hyperproteinorachia—indicating severe disruption of the blood–brain barrier and active bacterial replication. MRI excluded parenchymal lesions but demonstrated mastoid opacification, supporting an extracerebral focus of the infection. Hyperlactatemia likely reflects sepsis-related tissue hypoperfusion, further confirming a systemic process evolving in parallel with CNS invasion.

The patient’s comorbidities substantially increased his vulnerability to invasive infections. Type 2 diabetes mellitus and dyslipidemia impair innate immune responses and phagocytic function, while previous cerebrovascular events and pulmonary embolism indicate systemic endothelial dysfunction that may compromise the integrity of the blood–brain barrier (BBB). Recurrent urinary tract infections with multidrug-resistant Pseudomonas a. further exemplify impaired host–pathogen defense mechanisms. Collectively, these factors established a high-risk context in which a relatively minor ENT procedure precipitated fulminant and invasive disease [6].

A careful synthesis of the clinical course, systemic inflammatory markers, neuroimaging, and CSF findings suggests an unusually fulminant disease process. The rapid onset of fever, agitation, and encephalopathy within 48 h required consideration of both contiguous spread from the sinonasal or mastoid regions and hematogenous seeding in the setting of transient postoperative immune dysfunction. Imaging demonstrated mastoid involvement without parenchymal lesions, supporting contiguous extension, but the patient’s history of poorly controlled diabetes and recurrent urinary tract infections raises the possibility that transient bacteremia also contributed. In this patient, the most likely mechanism of infection was contiguous spread from the sinonasal and mastoid structures, as suggested by the MRI evidence of mastoid opacification. Nevertheless, transient postoperative bacteremia cannot be fully excluded, particularly considering the patient’s metabolic vulnerability and history of recurrent urinary tract infections. Taken together, these findings support a multifactorial pathogenesis involving both local disruption of sinonasal barriers and systemic immune compromise in patients with CRS.

CSF analysis demonstrated the classical triad of bacterial meningitis: marked neutrophilic pleocytosis, hypoglycorrhachia (CSF glucose 0 mg/dL), and markedly elevated protein (283 mg/dL) levels, with elevated opening pressure (200 mmH_2_O). These findings indicate substantial meningeal inflammation, with ongoing bacterial replication and blood–CSF barrier disruption. The profile is most consistent with established intrathecal infection rather than transient bacteremia alone, as profound hypoglycorrhachia and very high protein levels typically reflect sustained bacterial metabolism and inflammatory protein leakage.

Regional Romanian surveillance data (ECDC 2023) indicate penicillin non-susceptibility in approximately 16% and macrolide resistance in 23% of *S. pneumoniae* isolates, findings that closely mirror the resistance profile observed in this patient. At the broader European level, penicillin resistance rates approach 25%, with combined macrolide/β-lactam resistance reported in 10–15% of the invasive isolates. This case reflects both local and global antimicrobial resistance trends, highlighting the declining efficacy of traditional β-lactams and reinforcing the need for empiric regimens with reliable central nervous system penetration [7].

More broadly, surveillance across Europe and Asia has reported penicillin non-susceptible pneumococci in up to 25% of invasive isolates, with combined macrolide/β-lactam resistance in 10–15% [8]. These data emphasize the growing global challenge of AMR in pneumococcus, which complicates empirical therapy and underscores the importance of local antibiograms and rigorous surveillance [9].

The therapeutic choice of meropenem plus vancomycin provides broad coverage with reliable CNS penetration, which is appropriate for MDR pneumococcal meningitis. Vancomycin achieves bactericidal CSF concentrations when the meninges are inflamed, whereas meropenem retains activity against most penicillin-resistant strains [10,11]. De-escalation was not pursued, given the limited susceptibility to alternative agents. Adjunctive dexamethasone, administered with the first antibiotic dose, is supported by the IDSA and WHO guidelines and likely contributed to the absence of neurologicalsequelae [12,13]. This case highlights the importance of initiating broad-spectrum, high-barrier antimicrobials early in suspected cases of postoperative meningitis, with subsequent tailoring based on culture results.

Supportive care is essential in addition to antimicrobial therapy. Profound metabolic acidosis, hyperlactatemia, and transaminase elevation reflect systemic inflammatory response syndrome (SIRS) and tissue hypoperfusion. Hemodynamic optimization, electrolyte correction, and mechanical ventilation restored physiological balance, while antifungal therapy (fluconazole) and urinary antibiotics addressed ICU-related opportunistic infections. Successful recovery underscores that the outcomes of severe pneumococcal meningitis depend on both pathogen-targeted therapy and meticulous host-directed supportive measures.

Favorable neurological recovery after 10 days of critical care emphasizes the value of multidisciplinary teamwork involving intensivists, infectious disease specialists, neurologists, and ENT surgeons. Nonetheless, meningitis survivors remain at risk for late sequelae, including vestibular dysfunction, subtle cognitive deficits and psychosocial distress [14,15]. Therefore, structured follow-up, including neuropsychological testing and audiometry, should be integrated into post-discharge pathways [15,16].

A striking preventive gap in this case was the absence of pneumococcal vaccination. Contemporary guidelines from the CDC, ECDC, and multiple national societies recommend sequential PCV15 or PCV20 vaccination followed by PPSV23 vaccination in adults with diabetes, cardiovascular disease, or other chronic conditions [17]. Preoperative assessment represents an ideal opportunity to review the immunization status. Observational studies have shown a 50–70% reduction in IPD among vaccinated high-risk adults, including protection against MDR serotypes [17]. In this patient, vaccination administered at least two weeks before surgery could have prevented the invasive disease.

Therefore, we propose incorporating vaccine verification into pre-anesthesia checklists for high-risk surgical candidates. Operationalizing these recommendations in surgical practice requires their integration into standardized, preoperative protocols. Practical strategies include documenting vaccination status as part of the pre-anesthesia checklist, establishing same-day vaccination services within preoperative assessment units, and scheduling elective surgery at least two weeks after vaccine administration to ensure an adequate immune response. Implementing such measures would help shift pneumococcal vaccination from an optional consideration to a routine component of perioperative care [7].

Based on the CDC and ECDC recommendations, pneumococcal vaccination should ideally be administered at least two weeks before elective ENT surgery to allow an adequate immune response. For vaccine-naïve patients, a single dose of PCV15 or PCV20 is recommended, followed by PPSV23 one year later if PCV15 was used. In those previously vaccinated with PPSV23, conjugate vaccination should be delayed for at least one year. This timing ensures optimal protection against invasive pneumococcal disease and may reduce postoperative CNS infections.

A notable limitation of this study is the absence of serotyping for the isolate, leaving it unclear whether the strain is included in the protection offered by the current conjugate vaccines (PCV13, PCV15, and PCV20). Despite this uncertainty, the patient was administered PCV15 after discharge and was set to receive PPSV23 one year later, following CDC guidelines. This sequential vaccination strategy is intended to provide T-cell–mediated immunity through the conjugate vaccine and extend polysaccharide coverage to additional serotypes not addressed by PCV15.

Preventive measures extend beyond vaccination. Sinonasal surgery introduces a transient “window of vulnerability” characterized by mucosal disruption and altered local microbiota. Risk-reduction strategies should include meticulous aseptic techniques, limiting operative time, and patient education about early warning signs of infection. ENT surgeons should maintain a low threshold for reassessing patients with unexplained postoperative fever, delirium, or neurological symptoms. Selective screening of high-risk patients for pneumococcal colonization could inform tailored prophylaxis, although further evidence is required [18].

This case report had several limitations. First, pneumococcal serotyping was not performed, preventing the determination of whether the isolate was vaccine-preventable. Second, the exact route of infection could not be definitively established, although imaging and clinical findings suggested contiguous spread. Third, the generalizability of this study is limited by its single-case design. Future studies incorporating serotyping and genomic analyses are essential to better elucidate the epidemiology and preventability of similar cases.

Pharmacokinetic–pharmacodynamic modeling can refine the dosing of CNS-penetrating antibiotics in critically ill or obese patients [19]. Finally, routine serotyping of pneumococcal isolates would improve the understanding of virulence factors and epidemiology; however, it was not performed in this case, representing a limitation.

## 4. Conclusions

This case documents a fulminant episode of postoperative meningoencephalitis due to MDR *S. pneumoniae* after septoturbinoplasty, an exceptionally rare but life-threatening complication. The resistance profile of the isolate reflects the increasing global burden of pneumococcal antimicrobial resistance, underscoring the importance of rapid recognition, prompt initiation of broad-spectrum agents with reliable CNS penetration, and guidance from local antibiogram data.

The preventive dimension is equally important. Our patient, despite multiple comorbidities predisposing him to invasive pneumococcal disease, did not receive pneumococcal vaccination. This represents a critical missed opportunity for future research. Current CDC and ECDC recommendations emphasize the role of conjugate pneumococcal vaccines, ideally administered at least two weeks before elective surgery, in protecting high-risk adults against invasive disease.

Together, these observations highlight two complementary priorities for clinical practice: vigilance and preparedness to manage MDR pneumococcal infections in the perioperative setting and systematic incorporation of pneumococcal vaccination into preoperative assessments of high-risk surgical candidates. By addressing both therapeutic and preventive aspects, the morbidity and mortality of invasive pneumococcal disease in surgical populations may be substantially reduced.

## Figures and Tables

**Table 1:** Laboratory Investigations on Admission (24 March 2025).

Parameter	Result	Reference Range	Notes
**C-Reactive Protein (CRP)**	28.55 mg/L	<5 mg/L	Elevated inflammatory marker
**White Blood Cell Count (WBC)**	34.6 × 10^9^/L	4.0–11.0 × 10^9^/L	Neutrophilia and lymphopenia
**Arterial pH**	7.22	7.35–7.45	Metabolic acidosis
**Bicarbonate (HCO_3_)**	17.2 mmol/L	22–28 mmol/L	Metabolic acidosis
**Alanine Aminotransferase (ALT)**	73 U/L	7–56 U/L	Mildly elevated liver enzyme
**Lactate Dehydrogenase (LDH)**	402 U/L	140–280 U/L	Indicates tissue damage
**Creatine Kinase (CK)**	193 U/L	38–174 U/L	Muscle injury marker
**CK-MB**	34 U/L	<25 U/L	Cardiac muscle injury marker
**Sodium (Na^+^)**	120 mmol/L	135–145 mmol/L	Electrolyte imbalance
**Magnesium (Mg^2+^)**	1.4 mg/dL	1.7–2.2 mg/dL	Electrolyte imbalance
**Ferritin**	15 ng/mL	20–300 ng/mL	Hypoferritinemia
**Blood glucose**	172 mg/dL	70–99 mg/dL (3.9–5.5 mmol/L)	Hyperglycemia; consistent with uncontrolled diabetes

**Table 2:** Arterial Blood Gas (ABG) Analysis on Admission (24 March 2025).

Parameter	Result	Reference Range	Interpretation
pH	7.22	7.35–7.45	**Acidosis**
pCO_2_ (partial pressure of CO_2_)	37 mmHg	35–45 mmHg	Normal
HCO_3_^−^ (bicarbonate)	17.2 mmol/L	22–28 mmol/L	**Metabolic acidosis**
pO_2_ (partial pressure of O_2_)	78 mmHg	75–100 mmHg	Mild hypoxemia (on room air)
SaO_2_ (oxygen saturation)	94%	94–100%	Borderline
Lactate	5.7 mmol/L	<2.0 mmol/L	Suggests tissue hypoperfusion
Base Excess	−6 mmol/L *	−2 to +2 mmol/L	Consistent with metabolic acidosis

* A negative base excess indicates a deficit of base (bicarbonate) in the blood, reflecting metabolic acidosis. A magnitude of −6 mmol/L suggests a moderate metabolic component, often due to increased acid production (e.g., lactic acidosis) or bicarbonate loss.

**Table 3:** Cerebrospinal Fluid (CSF) Analysis.

Parameter	Result	Reference Range	Interpretation
Appearance	Opalescent	Clear	Typical of bacterial meningitis
Opening Pressure	200 mmH_2_O	70–180 mm H_2_O	Increased intracranial pressure
Cell Count (absolute)	1850 cells/mm^3^	<5 cells/mm^3^	Marked pleocytosis
Glucose	0	40–70 mg/dL	Indicative of bacterial consumption
Sodium	143 mmol/L	135–150 mmol/L	Within normal CSF sodium range
Potasium	2.90 mmol/L	2–3 mmol/L	Lower end of normal
Albumin	83 mg/dL	10–30 mg/dL	Elevated; suggests blood-CSF barrier dysfunction
Protein	283 mg/dL	15–45 mg/dL	Blood-CSF barrier disruption
Microbiology	*S. pneumoniae* isolated	Negative for other pathogens	Confirms pneumococcal infection

**Table 4:** Imaging Findings.

Imaging Modality	Date	Findings	Interpretation
CT of the brain	24 March 2025	No acute CNS lesions; mild ethmoidal/sphenoidal sinus opacification	No acute brain pathology; sinus inflammation
CT of the thorax and abdomen	24 March 2025	Hepatic steatosis	Chronic liver pathology
MRI of the brain	3 April 2025	Para-fluid collections in left mastoid air cells extending to Citelli’s angle; no parenchymal lesions or venous thrombosis	Mastoid air cell involvement; no brain abscess or thrombosis

## Data Availability

The original contributions presented in the study are included in the article; further inquiries can be directed to the corresponding author.
